# Halogen-bonded co-crystal containing 1,3-di­iodo­perchloro­benzene and the photoproduct *rtct*-tetra­kis­(pyridin-4-yl)cyclo­butane resulting in a zigzag topology

**DOI:** 10.1107/S2056989023001408

**Published:** 2023-02-21

**Authors:** Eric Bosch, Daniel K. Unruh, Carlos L. Santana, Ryan H. Groeneman

**Affiliations:** a Missouri State University, Department of Chemistry and Biochemistry, Springfield, MO 65897, USA; b Texas Tech University, Department of Chemistry and Biochemistry, Lubbock, TX 79409, USA; c Webster University, Department of Biological Sciences, St. Louis, MO 63119, USA; Purdue University, USA

**Keywords:** halogen bonding, organic solid state, co-crystal, photoproduct, cyclo­butane, [2 + 2] cyclo­addition reaction, zigzag network

## Abstract

The formation and crystal structure of a zigzag network held together by I⋯N halogen bonds is reported. In particular, the halogen-bond donor is 1,3-di­iodo­perchloro­benzene (**C_6_I_2_Cl_4_**) while the acceptor is the photoproduct *rtct*-tetra­kis­(pyridin-4-yl)cyclo­butane (**TPCB**). Curiously, within the resulting co-crystal (**C_6_I_2_Cl_4_**)·(**TPCB**), the photoproduct accepts only two halogen bonds between neighbouring 4-pyridyl rings and as a result behaves as a bent two-connected node rather than the expected four-connected centre. In addition, the photoproduct, **TPCB**, is also found to engage in C—H⋯N hydrogen bonds, forming an extended zigzag chain.

## Abstract 2

The formation of a co-crystal with a zigzag topology sustained by halogen bonds involving the photoproduct *rtct*-tetra­kis­(pyridin-4-yl)cyclo­butane acting as a two connecting node is reported.

## 1. Chemical context

A continued focus for crystal engineers is the formation of mol­ecular networks held together by non-covalent inter­actions (Vantomme & Meijer, 2019 ▸). Still today, research on these purely organic materials continues to lag behind related areas such as metal–organic frameworks as well as supra­molecular coordinated solids. Co-crystallization has proven to be a successful approach in the formation of these extended organic solids (Gunawardana & Aakeröy, 2018 ▸). As in all types of network design, the components of these co-crystals must be carefully considered to ensure complementary supra­molecular donor and acceptor sites that will allow for self-assembly of the multi-component solid. A highly utilized and well-established non-covalent inter­action is halogen bonding, which is defined as the inter­action between an electrophilic region on a halogen atom and a nucleophilic region on a different atom (Gilday *et al.*, 2015 ▸). Overall, the strength and directionality of halogen bonds makes them an ideal supra­molecular inter­action, along with hydrogen bonds, as a driving force in the formation of co-crystals (Corpinot & Bučar, 2019 ▸).

A continued area of focus between our research groups has been in the design and formation of halogen-bonded mol­ecular networks (Dunning *et al.*, 2021 ▸, 2022 ▸; Oburn *et al.*, 2020 ▸; Sinnwell *et al.*, 2020 ▸) that contain cyclo­butane-based nodes generated from the [2 + 2] cyclo­addition reaction between alkenes (Kole & Mir, 2022 ▸; Gan *et al.*, 2018 ▸). Recently, we reported the ability to form a mol­ecular salt with a square network topology based upon the tetra­protonated photoproduct *rtct*-tetra­kis­(pyridin-4-yl)cyclo­butane and the sulfate anion (Santana *et al.*, 2021*b* ▸). The *rtct*-isomer, which is the more stable thermodynamic product, is not directly observed from the solid-state [2 + 2] cyclo­addition reaction, but rather forms after the photoreaction and an acid-catalysed isomerization reaction (Hill *et al.*, 2012 ▸; Peedikakkal *et al.*, 2010 ▸). 
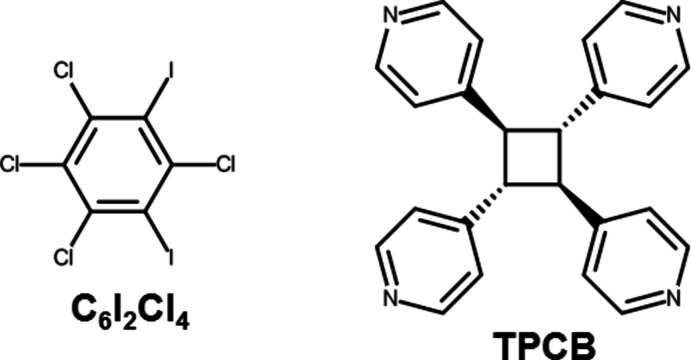


Herein, we report the solid-state crystal structure of a co-crystal held together by I⋯N halogen bonds that has a zigzag topology. In particular, the solid is based upon 1,3-di­iodo­perchloro­benzene (**C_6_I_2_Cl_4_**) acting as the halogen-bond donor while the photoproduct *rtct*-tetra­kis­(pyridin-4-yl)cyclo­butane (**TPCB**) behaves as the acceptor. Unexpectedly, the **TPCB** mol­ecule is found to accept only two I⋯N halogen bonds, between neighbouring 4-pyridyl rings, which makes the photoproduct act as a bent two-connected node rather than a four-connected node as seen in the square network topology with the sulfate anion (Santana *et al.*, 2021*b* ▸).

## 2. Structural commentary

**Figure 1:**
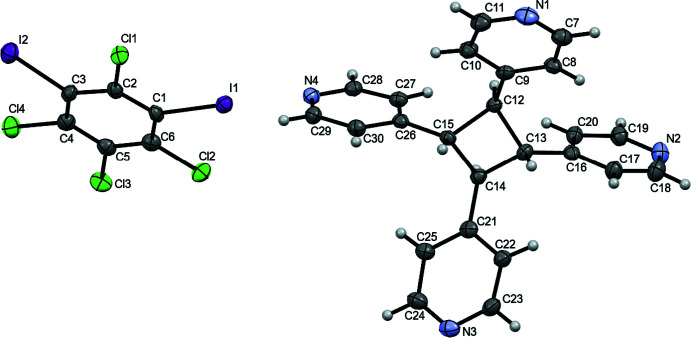
The labelled asymmetric unit of (**C_6_I_2_Cl_4_**)*·*(**TPCB**). Displacement ellipsoids are drawn at the 50% probability level for non-hydrogen atoms while hydrogen atoms are shown as spheres of arbitrary size.

**Figure 2:**
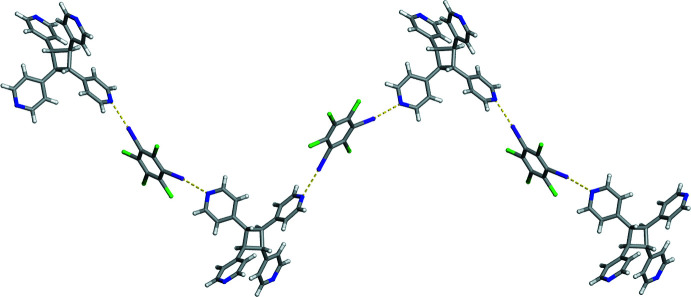
X-ray crystal structure of (**C_6_I_2_Cl_4_**)*·*(**TPCB**) illustrating the zigzag network held together by I⋯N halogen bonds. Halogen bonds are represented by yellow dashed lines.

Crystallographic analysis revealed that the components of (**C_6_I_2_Cl_4_**)*·*(**TPCB**) crystallize in the centrosymmetric monoclinic space group *P*2_1_/*c*. The asymmetric unit contains a full mol­ecule of both **C_6_I_2_Cl_4_** and **TPCB** (Fig. 1 ▸) although the crystals formed from a 2:1 solution of the two components. Notably, the **TPCB** mol­ecule has an *rtct*-geometry, as expected since we first subjected the *rctt*-**TPCB** to an acid-catalysed isomerization. As a result of the isomerization reaction, the bond angles between neighbouring 4-pyridyl rings within **TPCB** are nearly perpendicular, with all four angles slightly obtuse at 93.64 (7), 96.05 (7), 96.37 (7) and 100.50 (7)°. These bond angles were measured from the centroids of the cyclo­butane and the pyridine rings. As expected, the halogen-bond donor **C_6_I_2_Cl_4_** forms two crystallographically unique I⋯N halogen bonds with **TPCB**. The halogen-bond distances between I1⋯N4 and I2⋯N3^i^ have values of 2.757 (4) and 2.909 (4) Å along with bond angles for C1—I1⋯N4 and C3—I2⋯N3^i^ of 176.58 (15) and 172.73 (16)°, respectively [symmetry code: (i) 1 + *x*, 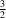 − *y*, − 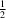 + *z*]. Surprisingly, within (**C_6_I_2_Cl_4_**)*·*(**TPCB**) only two adjacent 4-pyridyl rings are accepting these I⋯N halogen bonds. As a consequence of the observed formula and the lower than expected number of halogen bonds, **TPCB** behaves as a bent two-connecting node, resulting in a zigzag topology (Fig. 2 ▸). The pitch distance observed within (**C_6_I_2_Cl_4_**)*·*(**TPCB**) is 20.51 (2) Å measured from the centroids of two nearest cyclo­butane rings within the chain. Even though the I atoms on **C_6_I_2_Cl_4_** are found in the *meta* positions, rather than the *para* position, this halogen-bond donor acts as a nearly linear linker within (**C_6_I_2_Cl_4_**)*·*(**TPCB**) (Fig. 2 ▸).

## 3. Supra­molecular features

**Figure 3:**
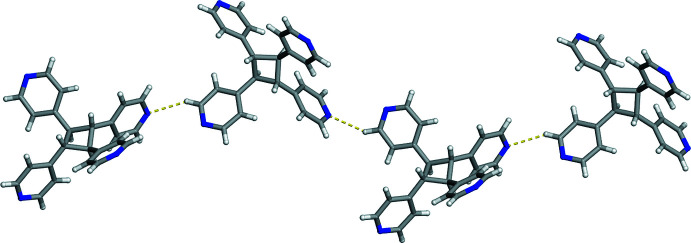
X-ray crystal structure of (**C_6_I_2_Cl_4_**)*·*(**TPCB**) illustrating the C—H⋯N hydrogen bonds between photoproducts forming a zigzag chain. Hydrogen bonds are represented by yellow dashed lines.

In addition to halogen bonding within (**C_6_I_2_Cl_4_**)*·*(**TPCB**), the photoproduct **TPCB** is found to engage in a C—H⋯N hydrogen bond, resulting in a mono-periodic zigzag chain (Fig. 3 ▸). In particular, this C—H⋯N hydrogen bond has a C⋯N separation of 3.442 (7) Å and a C—H⋯N angle of 148°. It is important to note that the hydrogen-bond-accepting N atom does not accept halogen bonds. The donor H atom for this C—H⋯N hydrogen bond is in the 3-position on a pyridine ring that accepts a halogen bond.

**Figure 4:**
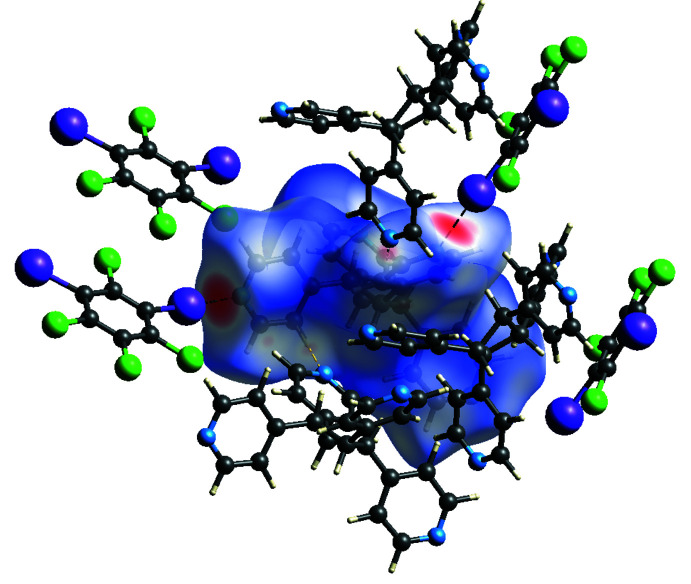
Hirshfeld surface of (**C_6_I_2_Cl_4_**)*·*(**TPCB**) mapped over *d*_norm_ illustrating the I⋯N halogen bonds (darkest red spots) and the C—H⋯N hydrogen bonds (faint red spots).

These different types of non-covalent inter­actions were also investigated and visualized by a Hirshfeld surface analysis (Spackman *et al.*, 2021 ▸) mapped over *d*_norm_ (Fig. 4 ▸). The darkest red spots on the Hirshfeld surface represent I⋯N halogen bonds while the faint red spots indicate the C—H⋯N inter­actions. The adjacent halogen bond accepting 4-pyridyl groups within **TPCB** generates the two-connecting node and the bent geometry required for a zigzag topology.

## 4. Database survey

A search of the Cambridge Crystallographic Database, Version 2022.3.0 Build 364, (Groom *et al.*, 2016 ▸) using *Conquest* (Bruno *et al.*, 2002 ▸) for structures containing tetra­kis­(pyridin-4-yl)cyclo­butane in which one pyridyl N atom is within the van der Waals radius of a halogen atom revealed a total of four structures. Two of these structures correspond to the *rtct*-isomer. One of these, refcode RULHAK, is our earlier report of the tetra­hedral network formed between 1,4-di­iodo­perchloro­benzene and *rctt*-**TPCB** (Oburn *et al.*, 2020 ▸), in which all four pyridyl N atoms are halogen-bond acceptors. In the other structure, refcode EKUJOM (Santana *et al.*, 2021*a* ▸), a chlorine atom *ortho* to a hydrogen-bonded phenol has a geometry-enforced close contact to the N atom.

## 5. Synthesis and crystallization

*Materials and general methods* The solvents such as reagent grade ethanol, dimethyl sulfoxide, chloro­form, and toluene were all purchased from Sigma-Aldrich Chemical (St. Louis, MO, USA) and used as received. In addition, resorcinol (**res**), *trans*-1,2-bis­(pyridin-4-yl)ethyl­ene (**BPE**), concentrated sulfuric acid, and sodium hydroxide pellets were also purchased from Sigma-Aldrich and were used without additional purification. The [2 + 2] cyclo­addition reaction was conducted in an ACE Glass photochemistry cabinet using UV-radiation from a 450 W medium-pressure mercury lamp. The occurrence of both the [2 + 2] cyclo­addition reaction along with the acid-catalysed isomerization reaction were confirmed by using ^1^H Nuclear Magnetic Resonance Spectroscopy on a Bruker Avance 400 MHz spectrometer with dimethyl sulfoxide (DMSO-*d*_6_) as the solvent. The halogen-bond donor 1,3-di­iodo­perchloro­benzene (**C_6_I_2_Cl_4_**) was synthesized utilizing a previously published method (Reddy *et al.*, 2006 ▸).

*Synthesis and crystallization* The formation of the halogen-bond acceptor *rtct*-tetra­kis­(pyridin-4-yl)cyclo­butane (**TPCB**) was achieved by using a previously published approach (Santana *et al.*, 2021*a* ▸). In particular, the co-crystal 2(**res**)*·*2(**BPE**) undergoes a [2 + 2] cyclo­addition reaction to yield *rctt*-tetra­kis­(pyridin-4-yl)cyclo­butane as previously reported (MacGillivray *et al.*, 2000 ▸). The *rctt*-photoproduct was removed from the template by means of a base extraction with 0.2 *M* sodium hydroxide solution along with chloro­form as the solvent. The conversion from *rctt*- to the *rtct*-isomer was achieved by heating 100 mg of the *rctt*-photoproduct in a 10 mL beaker with 2.0 mL of dimethyl sulfoxide along with two drops of sulfuric acid. The resulting solution was heated on a hot plate for one hour at 373 K (Peedikakkal *et al.*, 2010 ▸). The complete upfield shift of the cyclo­butane in the ^1^H NMR spectra from 4.86 ppm for the *rctt*-isomer to 3.86 ppm for the *rtct*-isomer confirms the qu­anti­tative yield for this isomerization reaction. The separation of the photoproduct from the sulfate salt was achieved by a base extraction with 0.2 *M* sodium hydroxide and again chloro­form in three 10.0 mL aliquots. Removal of the chloro­form yielded pure **TPCB** (Fig. 1 in the supporting information).

The formation of (**C_6_I_2_Cl_4_**)*·*(**TPCB**) was achieved by dissolving 64.0 mg of **C_6_I_2_Cl_4_** in 2.0 mL of toluene and then combined with a 2.0 mL ethanol solution containing 25.0 mg of **TPCB** (2:1 molar equivalent). Within three days, single crystals suitable for X-ray diffraction were formed upon loss of some of the solvent by slow evaporation.

## 6. Refinement

Crystal data, data collection and structure refinement details are summarized in Table 1 ▸. Intensity data were corrected for Lorentz, polarization, and background effects using *CrysAlis PRO* (Rigaku OD, 2021 ▸). A numerical absorption correction was applied based on a Gaussian integration over a multifaceted crystal and followed by a semi-empirical correction for absorption applied using the program *SCALE3 ABSPACK*. The program *SHELXT* (Sheldrick, 2015*a* ▸) was used for the initial structure solution while *SHELXL* (Sheldrick, 2015*b* ▸) for the refinement of the structure. Both programs were utilized within the *OLEX2* software (Dolomanov *et al.*, 2009 ▸). Hydrogen atoms bound to carbon atoms were located in the difference-Fourier map and were geom­etrically constrained using the appropriate AFIX commands.

## Supplementary Material

Crystal structure: contains datablock(s) I. DOI: 10.1107/S2056989023001408/zl5042sup1.cif

Structure factors: contains datablock(s) I. DOI: 10.1107/S2056989023001408/zl5042Isup2.hkl

NMR spectrum. DOI: 10.1107/S2056989023001408/zl5042sup3.docx

Supporting information file. DOI: 10.1107/S2056989023001408/zl5042Isup4.cml

CCDC reference: 2236158

Additional supporting information:  crystallographic information; 3D view; checkCIF report

## Figures and Tables

**Table 1:** Experimental details

Crystal data	
Chemical formula	C_24_H_20_N_4_·C_6_Cl_4_I_2_
*M*_r_	832.10
Crystal system, space group	Monoclinic, *P*2_1_/*c*
Temperature (K)	100
*a*, *b*, *c* (Å)	10.0779 (1), 31.1307 (3), 9.3360 (1)
β (°)	90.986 (1)
*V* (Å^3^)	2928.57 (5)
*Z*	4
Radiation type	Cu *K*α
μ (mm^−1^)	20.46
Crystal size (mm)	0.20 × 0.14 × 0.14

Data collection	
Diffractometer	XtaLAB Synergy, Dualflex, HyPix
Absorption correction	Gaussian (*CrysAlis PRO*; Rigaku OD, 2021 ▸)
*T*_min_, *T*_max_	0.077, 0.552
No. of measured, independent and observed [*I* > 2σ(*I*)] reflections	41005, 6078, 5833
*R*_int_	0.065
(sin θ/λ)_max_ (Å^−1^)	0.633

Refinement	
*R*[*F* ^2^ > 2σ(*F* ^2^)], *wR*(*F* ^2^), *S*	0.043, 0.115, 1.06
No. of reflections	6078
No. of parameters	361
H-atom treatment	H-atom parameters constrained
Δρ_max_, Δρ_min_ (e Å^−3^)	1.29, −1.59

Computer programs: *CrysAlis PRO* (Rigaku OD, 2021 ▸), *SHELXT2018/2* (Sheldrick, 2015*a* ▸), *SHELXL2018/3* (Sheldrick, 2015*b* ▸) and *OLEX2* (Dolomanov *et al.*, 2009 ▸).

## supplementary crystallographic information

### Crystal data

**Table:**

C_24_H_20_N_4_·C_6_Cl_4_I_2_	*F*(000) = 1608
*M**_r_* = 832.10	*D*_x_ = 1.887 Mg m^−^^3^
Monoclinic, *P*2_1_/*c*	Cu *K*α radiation, λ = 1.54184 Å
*a* = 10.0779 (1) Å	Cell parameters from 33737 reflections
*b* = 31.1307 (3) Å	θ = 2.8–77.2°
*c* = 9.3360 (1) Å	µ = 20.45 mm^−^^1^
β = 90.986 (1)°	*T* = 100 K
*V* = 2928.57 (5) Å^3^	Irregular, clear colourless
*Z* = 4	0.20 × 0.14 × 0.14 mm

### Data collection

**Table:**

XtaLAB Synergy, Dualflex, HyPix diffractometer	6078 independent reflections
Radiation source: micro-focus sealed X-ray tube, PhotonJet (Cu) X-ray Source	5833 reflections with *I* > 2σ(*I*)
Mirror monochromator	*R*_int_ = 0.065
Detector resolution: 10.0000 pixels mm^-1^	θ_max_ = 77.5°, θ_min_ = 2.8°
ω scans	*h* = −12→12
Absorption correction: gaussian (CrysAlisPro; Rigaku OD, 2021)	*k* = −39→37
*T*_min_ = 0.077, *T*_max_ = 0.552	*l* = −11→11
41005 measured reflections	

### Refinement

**Table:**

Refinement on *F*^2^	Primary atom site location: dual
Least-squares matrix: full	Hydrogen site location: inferred from neighbouring sites
*R*[*F*^2^ > 2σ(*F*^2^)] = 0.043	H-atom parameters constrained
*wR*(*F*^2^) = 0.115	*w* = 1/[σ^2^(*F*_o_^2^) + (0.0613*P*)^2^ + 12.119*P*] where *P* = (*F*_o_^2^ + 2*F*_c_^2^)/3
*S* = 1.06	(Δ/σ)_max_ = 0.001
6078 reflections	Δρ_max_ = 1.29 e Å^−^^3^
361 parameters	Δρ_min_ = −1.59 e Å^−^^3^
0 restraints	

### Special details

**Table:**

Geometry. All esds (except the esd in the dihedral angle between two l.s. planes) are estimated using the full covariance matrix. The cell esds are taken into account individually in the estimation of esds in distances, angles and torsion angles; correlations between esds in cell parameters are only used when they are defined by crystal symmetry. An approximate (isotropic) treatment of cell esds is used for estimating esds involving l.s. planes.

### Fractional atomic coordinates and isotropic or equivalent isotropic displacement parameters (Å^2^)

**Table:**

	*x*	*y*	*z*	*U*_iso_*/*U*_eq_	
I1	0.76302 (3)	0.50879 (2)	0.21577 (3)	0.02318 (10)	
I2	1.14818 (3)	0.64897 (2)	0.04465 (4)	0.02936 (11)	
Cl1	0.98841 (11)	0.58501 (4)	0.27061 (12)	0.0250 (2)	
Cl2	0.73773 (12)	0.49167 (4)	−0.14080 (13)	0.0281 (2)	
Cl3	0.88684 (12)	0.54236 (4)	−0.37433 (12)	0.0290 (2)	
Cl4	1.07924 (13)	0.61572 (4)	−0.28903 (13)	0.0327 (3)	
C1	0.8680 (4)	0.54128 (15)	0.0555 (5)	0.0204 (9)	
C2	0.9583 (4)	0.57339 (15)	0.0918 (5)	0.0209 (9)	
C3	1.0255 (5)	0.59739 (15)	−0.0116 (5)	0.0217 (9)	
C4	1.0010 (5)	0.58722 (16)	−0.1554 (5)	0.0247 (10)	
C5	0.9136 (5)	0.55427 (16)	−0.1959 (5)	0.0236 (9)	
C6	0.8470 (5)	0.53186 (16)	−0.0900 (5)	0.0225 (9)	
N1	0.6291 (5)	0.61957 (16)	−0.2002 (5)	0.0330 (10)	
N2	1.0786 (4)	0.73533 (15)	0.3749 (5)	0.0307 (9)	
N3	0.3362 (4)	0.78121 (14)	0.5891 (5)	0.0273 (9)	
N4	0.3606 (4)	0.53628 (13)	0.5702 (4)	0.0236 (8)	
C7	0.7200 (5)	0.64543 (17)	−0.1411 (6)	0.0281 (10)	
H7	0.780112	0.659565	−0.202606	0.034*	
C8	0.7319 (5)	0.65292 (16)	0.0056 (5)	0.0237 (9)	
H8	0.798244	0.671855	0.042088	0.028*	
C9	0.6457 (5)	0.63240 (15)	0.0980 (5)	0.0218 (9)	
C10	0.5535 (5)	0.60444 (16)	0.0385 (6)	0.0263 (10)	
H10	0.494626	0.588964	0.097843	0.032*	
C11	0.5480 (6)	0.59925 (18)	−0.1101 (6)	0.0307 (11)	
H11	0.483249	0.580267	−0.149585	0.037*	
C12	0.6517 (4)	0.64127 (14)	0.2565 (5)	0.0198 (9)	
H12	0.714761	0.620999	0.305369	0.024*	
C13	0.6797 (5)	0.68819 (15)	0.3058 (5)	0.0207 (9)	
H13	0.633720	0.708195	0.237435	0.025*	
C14	0.5868 (4)	0.67970 (15)	0.4330 (5)	0.0202 (9)	
H14	0.638296	0.666329	0.513896	0.024*	
C15	0.5189 (4)	0.64356 (15)	0.3393 (5)	0.0191 (9)	
H15	0.448070	0.656435	0.276453	0.023*	
C16	0.8187 (5)	0.70430 (15)	0.3320 (5)	0.0209 (9)	
C17	0.8615 (5)	0.74167 (17)	0.2657 (6)	0.0280 (10)	
H17	0.803580	0.757245	0.203397	0.034*	
C18	0.9901 (6)	0.75586 (18)	0.2917 (6)	0.0315 (11)	
H18	1.016950	0.781868	0.247481	0.038*	
C19	1.0362 (5)	0.69957 (17)	0.4377 (5)	0.0264 (10)	
H19	1.097139	0.684339	0.497523	0.032*	
C20	0.9090 (5)	0.68310 (16)	0.4216 (5)	0.0241 (9)	
H20	0.883834	0.657762	0.470998	0.029*	
C21	0.5012 (4)	0.71508 (15)	0.4889 (5)	0.0220 (9)	
C22	0.5111 (5)	0.75683 (16)	0.4375 (6)	0.0255 (10)	
H22	0.574784	0.763737	0.367313	0.031*	
C23	0.4277 (5)	0.78824 (16)	0.4892 (6)	0.0281 (10)	
H23	0.435614	0.816476	0.451741	0.034*	
C24	0.3279 (5)	0.74149 (18)	0.6397 (6)	0.0296 (11)	
H24	0.264454	0.735785	0.711315	0.036*	
C25	0.4073 (5)	0.70759 (16)	0.5935 (5)	0.0253 (10)	
H25	0.397271	0.679697	0.633192	0.030*	
C26	0.4656 (5)	0.60485 (15)	0.4143 (5)	0.0197 (9)	
C27	0.5467 (5)	0.57869 (15)	0.5005 (5)	0.0215 (9)	
H27	0.639630	0.583583	0.506637	0.026*	
C28	0.4896 (5)	0.54561 (15)	0.5768 (5)	0.0222 (9)	
H28	0.545384	0.528619	0.637202	0.027*	
C29	0.2839 (5)	0.56031 (16)	0.4856 (5)	0.0250 (10)	
H29	0.192019	0.553694	0.478941	0.030*	
C30	0.3309 (5)	0.59454 (16)	0.4064 (5)	0.0227 (9)	
H30	0.271976	0.610824	0.347239	0.027*	

### Atomic displacement parameters (Å^2^)

**Table:**

	*U*^11^	*U*^22^	*U*^33^	*U*^12^	*U*^13^	*U*^23^
I1	0.02362 (16)	0.02170 (16)	0.02419 (17)	−0.00011 (10)	−0.00097 (11)	0.00065 (10)
I2	0.02659 (17)	0.02932 (18)	0.03203 (18)	−0.00289 (12)	−0.00289 (13)	0.00211 (12)
Cl1	0.0244 (5)	0.0292 (6)	0.0214 (5)	−0.0035 (4)	−0.0020 (4)	−0.0017 (4)
Cl2	0.0307 (6)	0.0270 (6)	0.0265 (6)	−0.0042 (4)	−0.0058 (5)	−0.0025 (4)
Cl3	0.0320 (6)	0.0358 (6)	0.0192 (5)	0.0029 (5)	−0.0026 (4)	−0.0009 (4)
Cl4	0.0387 (7)	0.0331 (6)	0.0266 (6)	−0.0027 (5)	0.0065 (5)	0.0064 (5)
C1	0.020 (2)	0.022 (2)	0.019 (2)	0.0017 (17)	0.0051 (17)	0.0050 (17)
C2	0.017 (2)	0.023 (2)	0.023 (2)	0.0023 (17)	−0.0003 (17)	0.0022 (17)
C3	0.020 (2)	0.019 (2)	0.026 (2)	−0.0025 (16)	−0.0021 (18)	−0.0001 (17)
C4	0.025 (2)	0.024 (2)	0.025 (2)	0.0029 (18)	0.0005 (19)	0.0030 (18)
C5	0.022 (2)	0.027 (2)	0.022 (2)	0.0053 (18)	−0.0003 (18)	0.0024 (18)
C6	0.022 (2)	0.025 (2)	0.021 (2)	0.0045 (17)	−0.0042 (17)	0.0017 (18)
N1	0.038 (2)	0.037 (3)	0.023 (2)	0.005 (2)	−0.0017 (18)	−0.0079 (18)
N2	0.027 (2)	0.036 (2)	0.030 (2)	−0.0076 (18)	−0.0003 (18)	0.0024 (18)
N3	0.0214 (19)	0.028 (2)	0.032 (2)	0.0048 (16)	−0.0061 (17)	−0.0074 (17)
N4	0.028 (2)	0.022 (2)	0.0208 (19)	0.0000 (16)	0.0049 (16)	−0.0003 (15)
C7	0.032 (3)	0.028 (3)	0.024 (2)	0.005 (2)	0.002 (2)	−0.0005 (19)
C8	0.022 (2)	0.026 (2)	0.023 (2)	0.0006 (18)	0.0019 (18)	−0.0057 (18)
C9	0.023 (2)	0.020 (2)	0.023 (2)	0.0046 (17)	−0.0008 (18)	−0.0027 (17)
C10	0.025 (2)	0.027 (2)	0.027 (2)	−0.0009 (19)	−0.0014 (19)	−0.0063 (19)
C11	0.033 (3)	0.031 (3)	0.028 (3)	−0.001 (2)	−0.002 (2)	−0.006 (2)
C12	0.019 (2)	0.018 (2)	0.023 (2)	0.0008 (16)	−0.0005 (17)	−0.0028 (17)
C13	0.023 (2)	0.018 (2)	0.021 (2)	−0.0014 (17)	−0.0016 (17)	0.0011 (17)
C14	0.020 (2)	0.019 (2)	0.022 (2)	−0.0003 (16)	−0.0013 (17)	−0.0009 (17)
C15	0.017 (2)	0.021 (2)	0.020 (2)	0.0015 (16)	−0.0003 (17)	−0.0016 (17)
C16	0.021 (2)	0.024 (2)	0.018 (2)	−0.0017 (17)	0.0014 (17)	−0.0050 (17)
C17	0.027 (2)	0.026 (2)	0.032 (3)	−0.0012 (19)	0.001 (2)	0.004 (2)
C18	0.034 (3)	0.029 (3)	0.031 (3)	−0.006 (2)	0.001 (2)	0.004 (2)
C19	0.023 (2)	0.030 (3)	0.026 (2)	−0.0026 (19)	−0.0021 (19)	−0.0007 (19)
C20	0.025 (2)	0.025 (2)	0.022 (2)	−0.0043 (18)	0.0001 (18)	0.0004 (18)
C21	0.019 (2)	0.025 (2)	0.022 (2)	0.0010 (17)	−0.0064 (17)	−0.0031 (18)
C22	0.026 (2)	0.022 (2)	0.029 (2)	−0.0006 (18)	0.0001 (19)	−0.0024 (19)
C23	0.027 (2)	0.020 (2)	0.036 (3)	−0.0011 (18)	−0.004 (2)	−0.001 (2)
C24	0.025 (2)	0.033 (3)	0.030 (3)	0.005 (2)	0.001 (2)	−0.006 (2)
C25	0.026 (2)	0.025 (2)	0.024 (2)	0.0030 (19)	0.0011 (19)	−0.0022 (18)
C26	0.021 (2)	0.021 (2)	0.018 (2)	0.0007 (17)	0.0014 (17)	−0.0038 (16)
C27	0.022 (2)	0.023 (2)	0.020 (2)	0.0022 (17)	−0.0022 (17)	−0.0026 (17)
C28	0.025 (2)	0.021 (2)	0.021 (2)	0.0011 (17)	0.0006 (18)	−0.0026 (17)
C29	0.023 (2)	0.024 (2)	0.028 (2)	−0.0020 (18)	0.0007 (19)	−0.0029 (19)
C30	0.022 (2)	0.023 (2)	0.023 (2)	0.0022 (17)	−0.0024 (18)	0.0005 (18)

### Geometric parameters (Å, º)

**Table:**

I1—C1	2.106 (4)	C12—C15	1.558 (6)
I1—N4^i^	2.757 (4)	C13—H13	1.0000
I2—C3	2.088 (5)	C13—C14	1.548 (6)
I2—N3^ii^	2.909 (4)	C13—C16	1.504 (6)
Cl1—C2	1.730 (5)	C14—H14	1.0000
Cl2—C6	1.728 (5)	C14—C15	1.574 (6)
Cl3—C5	1.724 (5)	C14—C21	1.498 (6)
Cl4—C4	1.732 (5)	C15—H15	1.0000
C1—C2	1.390 (7)	C15—C26	1.498 (6)
C1—C6	1.402 (7)	C16—C17	1.390 (7)
C2—C3	1.404 (7)	C16—C20	1.392 (7)
C3—C4	1.397 (7)	C17—H17	0.9500
C4—C5	1.400 (7)	C17—C18	1.386 (8)
C5—C6	1.391 (7)	C18—H18	0.9500
N1—C7	1.332 (7)	C19—H19	0.9500
N1—C11	1.341 (8)	C19—C20	1.386 (7)
N2—C18	1.336 (7)	C20—H20	0.9500
N2—C19	1.332 (7)	C21—C22	1.390 (7)
N3—C23	1.340 (7)	C21—C25	1.391 (7)
N3—C24	1.327 (7)	C22—H22	0.9500
N4—C28	1.333 (6)	C22—C23	1.382 (7)
N4—C29	1.327 (7)	C23—H23	0.9500
C7—H7	0.9500	C24—H24	0.9500
C7—C8	1.392 (7)	C24—C25	1.397 (7)
C8—H8	0.9500	C25—H25	0.9500
C8—C9	1.391 (7)	C26—C27	1.398 (7)
C9—C10	1.383 (7)	C26—C30	1.397 (7)
C9—C12	1.506 (6)	C27—H27	0.9500
C10—H10	0.9500	C27—C28	1.383 (7)
C10—C11	1.397 (7)	C28—H28	0.9500
C11—H11	0.9500	C29—H29	0.9500
C12—H12	1.0000	C29—C30	1.385 (7)
C12—C13	1.556 (6)	C30—H30	0.9500
			
C1—I1—N4^i^	176.58 (15)	C21—C14—C13	120.1 (4)
C3—I2—N3^ii^	172.73 (16)	C21—C14—H14	109.7
C2—C1—I1	120.5 (3)	C21—C14—C15	118.1 (4)
C2—C1—C6	118.3 (4)	C12—C15—C14	86.5 (3)
C6—C1—I1	121.2 (4)	C12—C15—H15	109.8
C1—C2—Cl1	119.3 (4)	C14—C15—H15	109.8
C1—C2—C3	122.5 (4)	C26—C15—C12	120.9 (4)
C3—C2—Cl1	118.3 (4)	C26—C15—C14	118.1 (4)
C2—C3—I2	121.8 (4)	C26—C15—H15	109.8
C4—C3—I2	120.7 (4)	C17—C16—C13	120.1 (4)
C4—C3—C2	117.4 (4)	C17—C16—C20	117.4 (4)
C3—C4—Cl4	120.1 (4)	C20—C16—C13	122.5 (4)
C3—C4—C5	121.7 (4)	C16—C17—H17	120.5
C5—C4—Cl4	118.2 (4)	C18—C17—C16	119.0 (5)
C4—C5—Cl3	120.4 (4)	C18—C17—H17	120.5
C6—C5—Cl3	120.6 (4)	N2—C18—C17	124.2 (5)
C6—C5—C4	119.0 (5)	N2—C18—H18	117.9
C1—C6—Cl2	120.1 (4)	C17—C18—H18	117.9
C5—C6—Cl2	118.7 (4)	N2—C19—H19	117.8
C5—C6—C1	121.1 (5)	N2—C19—C20	124.4 (5)
C7—N1—C11	116.6 (5)	C20—C19—H19	117.8
C19—N2—C18	116.1 (5)	C16—C20—H20	120.6
C24—N3—C23	116.6 (4)	C19—C20—C16	118.9 (5)
C29—N4—C28	117.5 (4)	C19—C20—H20	120.6
N1—C7—H7	118.1	C22—C21—C14	121.5 (4)
N1—C7—C8	123.8 (5)	C22—C21—C25	116.9 (4)
C8—C7—H7	118.1	C25—C21—C14	121.6 (4)
C7—C8—H8	120.4	C21—C22—H22	120.2
C9—C8—C7	119.3 (5)	C23—C22—C21	119.6 (5)
C9—C8—H8	120.4	C23—C22—H22	120.2
C8—C9—C12	120.7 (4)	N3—C23—C22	123.9 (5)
C10—C9—C8	117.5 (5)	N3—C23—H23	118.1
C10—C9—C12	121.7 (4)	C22—C23—H23	118.1
C9—C10—H10	120.4	N3—C24—H24	118.2
C9—C10—C11	119.1 (5)	N3—C24—C25	123.7 (5)
C11—C10—H10	120.4	C25—C24—H24	118.2
N1—C11—C10	123.6 (5)	C21—C25—C24	119.3 (5)
N1—C11—H11	118.2	C21—C25—H25	120.3
C10—C11—H11	118.2	C24—C25—H25	120.3
C9—C12—H12	110.3	C27—C26—C15	121.8 (4)
C9—C12—C13	117.8 (4)	C30—C26—C15	121.1 (4)
C9—C12—C15	118.4 (4)	C30—C26—C27	117.0 (4)
C13—C12—H12	110.3	C26—C27—H27	120.4
C13—C12—C15	87.9 (3)	C28—C27—C26	119.1 (4)
C15—C12—H12	110.3	C28—C27—H27	120.4
C12—C13—H13	108.4	N4—C28—C27	123.5 (4)
C14—C13—C12	87.6 (3)	N4—C28—H28	118.2
C14—C13—H13	108.4	C27—C28—H28	118.2
C16—C13—C12	121.7 (4)	N4—C29—H29	118.3
C16—C13—H13	108.4	N4—C29—C30	123.5 (5)
C16—C13—C14	120.4 (4)	C30—C29—H29	118.3
C13—C14—H14	109.7	C26—C30—H30	120.4
C13—C14—C15	87.6 (3)	C29—C30—C26	119.3 (4)
C15—C14—H14	109.7	C29—C30—H30	120.4
			
I1—C1—C2—Cl1	2.5 (5)	C12—C13—C14—C21	−145.4 (4)
I1—C1—C2—C3	−176.7 (3)	C12—C13—C16—C17	127.3 (5)
I1—C1—C6—Cl2	−2.3 (5)	C12—C13—C16—C20	−52.6 (6)
I1—C1—C6—C5	178.0 (3)	C12—C15—C26—C27	45.7 (6)
I2—C3—C4—Cl4	3.3 (6)	C12—C15—C26—C30	−137.4 (5)
I2—C3—C4—C5	−176.5 (4)	C13—C12—C15—C14	−23.8 (3)
Cl1—C2—C3—I2	−4.6 (5)	C13—C12—C15—C26	−144.7 (4)
Cl1—C2—C3—C4	179.3 (4)	C13—C14—C15—C12	24.0 (3)
Cl3—C5—C6—Cl2	0.3 (6)	C13—C14—C15—C26	147.4 (4)
Cl3—C5—C6—C1	−180.0 (4)	C13—C14—C21—C22	−5.7 (7)
Cl4—C4—C5—Cl3	0.7 (6)	C13—C14—C21—C25	173.7 (4)
Cl4—C4—C5—C6	−178.2 (4)	C13—C16—C17—C18	179.9 (5)
C1—C2—C3—I2	174.6 (3)	C13—C16—C20—C19	178.6 (4)
C1—C2—C3—C4	−1.5 (7)	C14—C13—C16—C17	−125.0 (5)
C2—C1—C6—Cl2	179.0 (4)	C14—C13—C16—C20	55.1 (6)
C2—C1—C6—C5	−0.7 (7)	C14—C15—C26—C27	−58.1 (6)
C2—C3—C4—Cl4	179.5 (4)	C14—C15—C26—C30	118.8 (5)
C2—C3—C4—C5	−0.4 (7)	C14—C21—C22—C23	178.2 (4)
C3—C4—C5—Cl3	−179.5 (4)	C14—C21—C25—C24	−178.6 (4)
C3—C4—C5—C6	1.6 (7)	C15—C12—C13—C14	24.3 (3)
C4—C5—C6—Cl2	179.2 (4)	C15—C12—C13—C16	149.0 (4)
C4—C5—C6—C1	−1.1 (7)	C15—C14—C21—C22	−110.5 (5)
C6—C1—C2—Cl1	−178.8 (3)	C15—C14—C21—C25	68.9 (6)
C6—C1—C2—C3	2.0 (7)	C15—C26—C27—C28	174.5 (4)
N1—C7—C8—C9	0.4 (8)	C15—C26—C30—C29	−175.3 (4)
N2—C19—C20—C16	1.4 (8)	C16—C13—C14—C15	−149.8 (4)
N3—C24—C25—C21	0.1 (8)	C16—C13—C14—C21	88.8 (5)
N4—C29—C30—C26	0.0 (8)	C16—C17—C18—N2	1.7 (9)
C7—N1—C11—C10	1.0 (8)	C17—C16—C20—C19	−1.3 (7)
C7—C8—C9—C10	1.5 (7)	C18—N2—C19—C20	0.0 (8)
C7—C8—C9—C12	−177.2 (4)	C19—N2—C18—C17	−1.6 (8)
C8—C9—C10—C11	−2.2 (7)	C20—C16—C17—C18	−0.1 (7)
C8—C9—C12—C13	38.1 (6)	C21—C14—C15—C12	147.1 (4)
C8—C9—C12—C15	141.9 (5)	C21—C14—C15—C26	−89.5 (5)
C9—C10—C11—N1	0.9 (8)	C21—C22—C23—N3	0.7 (8)
C9—C12—C13—C14	145.5 (4)	C22—C21—C25—C24	0.8 (7)
C9—C12—C13—C16	−89.8 (5)	C23—N3—C24—C25	−0.6 (8)
C9—C12—C15—C14	−144.6 (4)	C24—N3—C23—C22	0.2 (8)
C9—C12—C15—C26	94.5 (5)	C25—C21—C22—C23	−1.2 (7)
C10—C9—C12—C13	−140.6 (5)	C26—C27—C28—N4	1.8 (7)
C10—C9—C12—C15	−36.8 (6)	C27—C26—C30—C29	1.7 (7)
C11—N1—C7—C8	−1.7 (8)	C28—N4—C29—C30	−0.9 (7)
C12—C9—C10—C11	176.6 (5)	C29—N4—C28—C27	−0.1 (7)
C12—C13—C14—C15	−24.0 (3)	C30—C26—C27—C28	−2.6 (6)

Symmetry codes: (i) −*x*+1, −*y*+1, −*z*+1; (ii) *x*+1, −*y*+3/2, *z*−1/2.
