# Supplementary material for: Halogen-bonded co-crystal containing 1,3-di­iodo­perchloro­benzene and the photoproduct rtct-tetra­kis­(pyridin-4-yl)cyclo­butane resulting in a zigzag topology
Source: Acta Crystallogr E Crystallogr Commun. 2023 Feb 21;79(Pt 3):212–5. doi: 10.1107/S2056989023001408 (PMC9993924; doi:10.1107/S2056989023001408)
Supplement: Supplementary file 3 [file e-79-00212-sup3.docx]

Supporting information

**Halogen-bonded co-crystal containing 1,3-diiodoperchlorobenzene and the photoproduct *rtct*-tetrakis(pyridin-4-yl)cyclobutane resulting in a zigzag topology**

**Eric Bosch, Daniel K. Unruh, Carlos L. Santana and Ryan H. Groeneman***


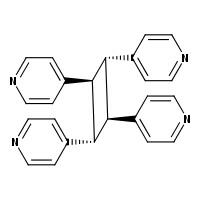


c

b

a

a

c

b

**Figure S1: ^1^**H NMR spectrum of *rtct*-tetrakis(pyridin-4-yl)cyclobutane (**TPCB**) (400 MHz, DMSO-*d*_6_).
